# Outdoor Kindergartens: A Structural Way to Improve Early Physical Activity Behaviour?

**DOI:** 10.3390/ijerph20065131

**Published:** 2023-03-14

**Authors:** Jeanett Friis Rohde, Sofus Christian Larsen, Mathilde Sederberg, Anne Bahrenscheer, Ann-Kristine Nielsen, Berit Lilienthal Heitmann, Ina Olmer Specht

**Affiliations:** 1 Research Unit for Dietary Studies, The Parker Institute, Bispebjerg and Frederiksberg Hospital, The Capital Region, Nordre Fasanvej 57, Vej 8, Entrance 11, 2000 Frederiksberg, Denmark; 2 Department of Pedagogy, Education Research and Development, Faculty of Education and Social Sciences, Copenhagen University College, Campus Hillerød, Carlsbergvej 14, 3400 Hillerød, Denmark; 3 The Boden Initiative, The Charles Perkins Centre, The University of Sydney, Sydney, NSW 2006, Australia; 4 Section for General Practice, Department of Public Health, University of Copenhagen, Øster Farimagsgade 5, 1353 Copenhagen, Denmark

**Keywords:** kindergarten, preschool children, physical activity, outdoor environment, nature, step counts

## Abstract

Background: Studies have shown that outdoor play in nature is associated with a higher physical activity level than indoor play. We aimed to examine the effect of outdoor versus conventional kindergartens on objectively measured physical activity. Method: Using a pre-test-post-test design, we collected data in four kindergartens that provided a rotating outdoor and conventional kindergarten setting. Step counts were measured during one week in the outdoor setting and one week in the conventional setting. Differences in step counts between the outdoor and conventional setting were analysed using a paired t-test. Results: In total, 74 children were included. There was no statistically significant difference in total daily step counts between children in the two settings. When we looked at step counts during kindergarten hours, we saw that children were more physically active in the outdoor setting compared to the conventional setting (mean difference: 1089, *p* < 0.0001). When we looked at activity during time outside the kindergarten, we discovered that children had a lower step count in the outdoor setting as compared to the conventional setting (mean difference −652, *p* = 0.01). Conclusion: This study indicates that children are more physically active during the time they spend in outdoor kindergartens compared to conventional kindergartens, but may compensate with more inactivity outside kindergarten hours.

## 1. Introduction

Several studies have shown that physically active children tend to remain more physically active across their lifespan [1]. Ideally, physical activity (PA) should be promoted among children of preschool age because it is easier to establish healthy behaviours in early childhood [2]. Nevertheless, a sedentary lifestyle with a long time spent indoors in front of a screen is becoming more and more common in children as young as three to five years, especially among children in urban areas [3]. This unhealthy lifestyle of preschoolers, leading to a low level of PA, is related to health problems including obesity and later increased risk of diabetes, cardiovascular diseases, musculoskeletal pain, and low self-esteem [4].

In Scandinavia, up to 97% of all children at the age of three to five years attend kindergartens [5], and around 67% of Danish children spends 6–8 h per day in kindergartens [6]. Therefore, the kindergarten environment presents an ideal setting to promote the early development of healthy PA behaviour [7]. Thus, kindergartens are an obvious place to create changes in children’s possibilities for physical experience and in relation to their PA level, motor skills, health, and possibly their well-being in the short and long term, regardless of social background, genes, and motor skills.

In Denmark and other Scandinavian countries, children can attend two types of kindergartens. A conventional kindergarten with time spent both indoors playing with toys, drawing etc., and with options for outdoor activities in the kindergarten playground, and an outdoor kindergarten, where children spend almost all the hours during the day outdoors in forests or in rural areas often without a formal playground and little if any option for indoor activities. A combination of the two types also exists, the rotating kindergarten, where children on a weekly basis change their kindergarten environment from the conventional kindergarten to the outdoor kindergarten [8,9,10].

It has been suggested that increasing outdoor playing among children aged three to twelve years is positively associated with PA and inversely associated with sedentary activity [11]. Several studies have shown that children playing outdoors had a higher PA level as compared to indoor play [7,12]. Additionally, previous cross-sectional studies have indicated that children playing in a forest environment seem to have a higher overall PA level than children playing in playgrounds [13,14]. The uneven ground and more room for ingenuity have been proposed as the factors explaining the higher PA level observed in children playing in forest environments [15,16]. However, only a few, small studies have attempted to investigate differences in PA between children attending outdoor kindergartens compared to those in conventional kindergartens [12]. In this regard, a Swedish cross-sectional study, conducted among 369 preschool children and 84 preschool teachers found that preschool structural characteristics such as PA policies and more time spent outdoors were positively associated with children’s PA behaviour [12]. Another cross-sectional study conducted among 864 preschool children in Finland showed that frequent nature trips were associated with lower sedentary activity during preschool hours [17]. These findings would indicate that more structured outdoor activity during kindergarten may increase PA and decrease sedentary behaviour. However, given the cross-sectional nature of the previously conducted studies [12,17,18,19], studies are needed that follow children over time and use an experimental research design.

Motor skill proficiency is known to be an important factor for future PA engagement and sports motivation [20]. Only a few studies have investigated motor abilities among children attending either an outdoor or a conventional kindergarten, and with mixed results. A Danish study which, among others, investigated motor development during 10 months among children in one outdoor kindergarten compared to a conventional kindergarten, showed that children in the outdoor kindergarten scored better in terms of attention, ingenuity and motor development. However, regarding frequency of illness, there was only a small difference between the groups [21]. Similarly, a Norwegian study among five-to-sevenyear-old kindergarten children that investigated versatile play during a nine-month period in an outdoor forest environment compared to a kindergarten playground, found that the children who daily played for 1–2 h in the forest gradually improved their motor ability more than the children who spent 1–2 h daily in the kindergarten playground [15]. However, in a large newly published study we did not see differences in motor abilities upon entrance to school between children attending either an outdoor or a conventional kindergarten [22].

In our previous study, we showed that children attending outdoor kindergartens differed in relation to parental socio- and early childhood demographics as compared to children attending conventional kindergartens, which can cause selection bias and is thus highly relevant to consider when investigating health outcomes related to kindergarten type attainment [23]. Thus, to avoid this, an ideal setting to investigate PA activity could be in rotating kindergartens, since the children here will be their own controls.

In this study we aimed to investigate whether children attending rotating kindergartens were more physically active while in the outdoor kindergarten setting compared to when they were in the conventional kindergarten setting.

## 2. Materials and Methods

The present study is part of the “Outdoor kindergartens-the healthier choice?“ (ODIN) project, which aimed to investigate short- and long-term health effects on children attending outdoor and conventional kindergartens.

### 2.1. Study Population

**Figure 1:**
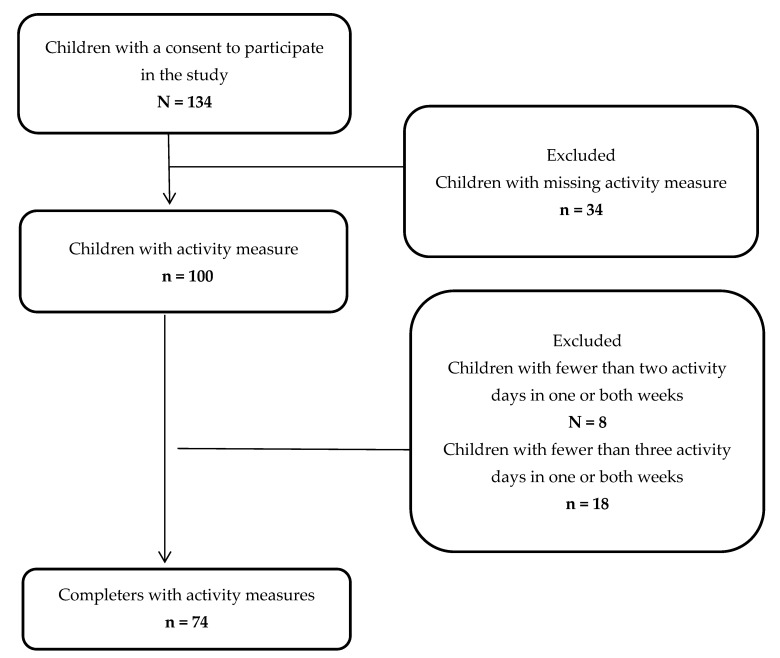
Flowchart of the study population.

In total, parents of 134 children aged two to six years signed a consent to participate in the study. In cases where the child did not want to wear the activity tracker, this was respected. Children with missing measurements on PA level, in some cases due to loss of activity measures or missing data due to full data storage of the activity measure (n = 34) and children with fewer than three activity days in one or both weeks were excluded (n = 26). The final study population for analysis consisted of 74 children (Figure 1).

### 2.2. Data Collection

Data collection for the present study was conducted in four rotating kindergartens in the Copenhagen area in the period February to mid-March 2020 and in September to December 2020. The data collection was paused due to the COVID-19 lockdown of all Danish kindergartens from 13 March 2020 until September 2020. The four rotating kindergartens both had an outdoor kindergarten setting and a conventional kindergarten setting, where the same group of children were one week in the outdoor setting and one week in the conventional setting. In that way, the children were their own controls.

### 2.3. Exposure Assessment

The exposure was the kindergarten setting (outdoor/conventional). Children spend one week in the outdoor setting and one week in the conventional setting consecutively, and the order of rotation was random between the four included kindergartens. The conventional kindergarten settings were kindergartens in Copenhagen city where children spend time both indoors playing with toys, drawing etc. and with options for outdoor activities in the kindergarten playground. Before lunch, the activities are structured by the kindergarten teachers and could be both inside or outside. After lunch, the children are most often sent outside (for approximately 2 h) to play in the playground (weather permitting). The children decide for themselves what they want to do; however, the kindergarten teachers also organize activities in the playground, which the children can choose to participate in.

The outdoor settings were outside Copenhagen in an environment surrounded by nature and where children spend almost full time outdoors in all seasons. Every morning parents dropped off the children at a collection point, typically the address of the conventional kindergarten setting, and from there the children were driven by bus for 30–60 min to the outdoor kindergarten setting. In the afternoon, the parents picked up the children at the drop-off point.

### 2.4. Outcome Assessment

To obtain information about the PA behaviours, the SENS motion^®^ system was used (https://sens.dk/da/, accessed on 1 February 2022). These sensors are medically approved devices designed for the long-term monitoring of physical activity. The sensors have only been validated among adults, but were chosen because of their ability to measure long-term physical activity [24,25]. The SENS motion^®^ system consists of a single-use miniature tri-axial accelerometer (dimensions 50  ×  21  ×  5 mm, weight 8 g; SENS motion^®^ activity measurement system) and a smartphone application. The system measures movement continuously at 12.5 Hz (every 10 s), 24 h a day. The accelerometer was placed on the lateral side of the right thigh on the first visit with a small waterproof band-aid (Medipore™, 3M, Soft Cloth Surgical Tape on Liner). The accelerometer was waterproof, and it was therefore not necessary to remove it during bathing and swimming. The accelerometer has an onboard memory of approximately 14 days.

Data from the accelerometer were uploaded to the app via Bluetooth and then transmitted to a secured web server for storage and subsequent analysis. To avoid loss of data (due to full memory), the preschool teachers or researchers were required to connect the accelerometer to the app at least once a week. During the study period, participants could change the band-aid if needed. An instruction sheet and additional band-aids were provided to the preschool teachers and parents. The system presents an overall count of steps per day and furthermore has a built-in algorithm that categorized data into seven categories: (1) lying or sitting, (2) standing, (3) walking, (4) sporadic walking, (5) running, (6) cycling and (7) no data. Data from the predefined activity categories will not be evaluated in this study because these measurements were not validated among the children.

The primary outcome, number of steps, was presented as an average of steps per day (Monday–Friday). Data were summarized as overall count of steps per day, count of steps taken during kindergarten hours (10:00 A.M. to 3:00 P.M., exclusive of bus transportation) and as count of steps taken outside kindergarten (time outside 10:00 A.M. to 3:00 P.M., including bus transportation).

Children wore the accelerometer continuously for 12 days (10 week days and two weekend days). Research suggested that three days was acceptable to monitor preschool children’s PA levels [26]. Therefore, for the present study children should at least have worn the accelerometer for a minimum of three days in each kindergarten. As wear time from previous studies among preschool children varied with regard to the definition of a valid day, with six and ten valid hours being the most common, we decided that children should at least have 10 h of wear time per day to be included in the analysis [27]. Six out of the 10 h should be registered between 8:00 A.M. and 5:00 P.M., with the assumption that this would be during the period when Danish kindergarten institutions are generally open.

### 2.5. Statistical Analyses

Descriptive statistics were presented as mean and standard deviations (SD) and percentages according to the type of outcome (binary/continual). Next, we used paired sample t-tests to analyse differences in PA behaviour between kindergarten settings (outdoor/conventional). We tested for differences in daily step counts during the whole day and separately for activity during kindergarten and leisure time.

Interaction between gender and kindergarten settings in relation to total daily steps counts was explored in a repeated measures ANCOVA model. Potential significant interaction was further evaluated through stratified analyses. Given that the present study was paused due to the initial Danish COVID-19 lockdown and that kindergartens were advised to let children spend most of the day outside post lockdown, we chose additionally to investigate possible effect modification between the enrolment date and the kindergarten setting in relation to total daily steps.

Moreover, analyses were performed to assess whether those children included in this study differed from those not included with regard to gender, ethnicity, transportation form to kindergarten, sports activities, whether parents were active with their child, whether the parents were participating in sports, and parental education levels.

All statistical analyses were two-sided with a significance level at 0.05 and were performed using Stata SE 14 (StataCorp LP, College Station, TX, USA; www.stata.com, accessed on 1 February 2022).

A power calculation was performed before the study began. Assuming a population of 40 children from outdoor kindergartens and 40 from conventional kindergartens, and a power of 80% and 5% significance, we would be able to detect a group difference of 3293 steps counts per day [28].

### 2.6. Ethics

Permission from the Ethical Committee was evaluated not to be relevant (journal nr.: H-19053587). Permission from the Capital Region Data Agency and the Danish Patient Safety Authority were granted (Journal nr.: P-2020-54 and 31-1521-8, respectively). Furthermore, parents and preschool teachers gave written consent for participation and for data to be used in the project. Information on full names, social security numbers or home addresses was not collected, and data were processed anonymized. Kindergarten teachers were informed about their right to withdraw at any time from the study. On behalf of the children, all parents were informed of their right to withdraw their children at any time from the study. In cases where the child did not want to wear the activity tracker, this was respected.

## 3. Results

In total, 74 children (55% out of 134) had sufficient PA measurements (a minimum of three days of activity per week) and were included for analyses. The children’s demographics are outlined in Table 1. Not all of the included children had information on demographic factors (Table 1). The mean age of the included children was five years (SD: one) and most children (>87%) were of Western origin. More girls (65.08%) than boys were included in the study.

**Figure 2:**
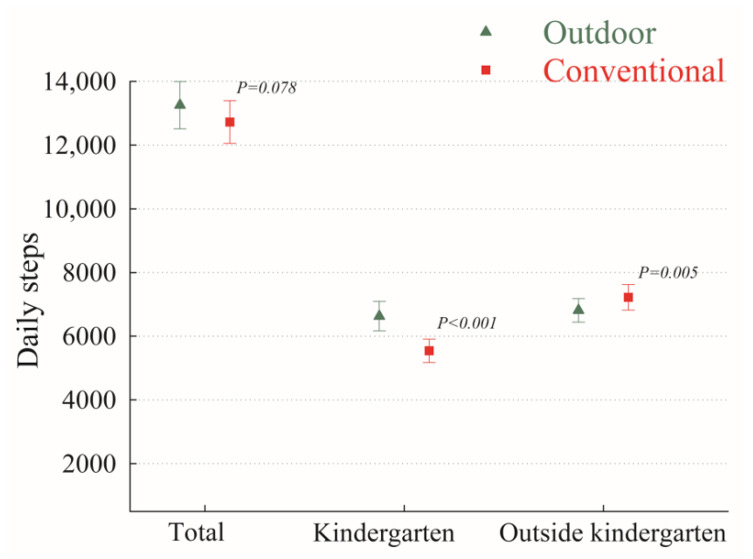
Differences in step counts (mean, 95% CI) between children in outdoor and conventional kindergarten setting (n = 74).

There were no statistically significant differences in the step counts per day between the outdoor setting (mean: 13,253; SD: 3205) and the conventional setting (mean: 12,726; SD: 2896) (mean difference 527, 95% CI: −61; 1115, *p* = 0.08) (Table 2). A difference in step counts was seen when we specifically looked at the time spent in the kindergarten, where children in the outdoor setting were more physically active as compared to children in the conventional setting (mean difference 1089, 95% CI: 669; 1508, *p* < 0.0001). However, a difference in step counts was seen when we specifically looked at the time spent outside the kindergarten, where children in the outdoor setting were less physically active as compared to children in the conventional setting (mean difference −562, 95% CI: −952; −171, *p* < 0.01) (Table 2, Figure 2).

We further found no evidence of an interaction between kindergarten setting (outdoor/conventional) and child gender in relation to total daily step counts (*p* = 0.14). Moreover, we found no evidence of effect modification by enrolment date (before/after the Danish COVID-19 lockdown) (*p* = 0.13).

Overall, the children included in this study were similar to those not included with respect to ethnicity, sporting activity, transportation form to/from kindergarten, whether the parents were active with their child, whether the parents participated in sports, and in relation to parental education level (Table 3). However, there was a higher proportion of girls among those included than those not included (*p* = 0.01) (Table 3).

## 4. Discussion

The current study examined whether children, on those days they attended an outdoor kindergarten setting, were more physically active compared to when they were in a conventional kindergarten setting. Our findings indicated that time spent in an outdoor kindergarten was directly associated with preschool children’s step counts during kindergarten hours, but had no statistically significant influence on total daily step counts. The week the children were in the outdoor setting, they were less active outside kindergarten hours compared to the week they were in the conventional setting.

The majority of earlier interventions on PA levels among preschoolers reported a small-to-moderate effect on general PA and a moderate effect on moderate-to-vigorous PA (MVPA) [7]. Those interventions with the greatest effects on MVPA that were short-term (i.e., lasted less than four weeks), were offered in childcare such as an early-learning environment, were led by teachers, involved outdoor activity, and incorporated unstructured activities [7]. Outdoor kindergartens include most of these elements, and as shown in the present study, children were more active during kindergarten hours when in the outdoor kindergarten setting compared to the conventional kindergarten setting. A recent systematic review highlights that pre-school environments with the presence of vegetation, open areas, portable play equipment, and lower playground density were positively associated with higher PA [29]. However, the review highlighted that more studies are needed to describe the association between environment and PA levels.

Because we had measures of all-day activity (24 h), it was possible to isolate activity during kindergarten hours and non-kindergarten hours. Our results indicated that some degree of subsequent compensation or more sedentary activity outside kindergarten time may occur. This was potentially due to transport to and from the outdoor setting or the possibility that time spent outside in the outdoor kindergarten may generate more fatigue in children. Spending a good part of the day outside exposed to environmental conditions may lead to children feeling more relaxed and less likely to go outside and/or be physically active after kindergarten. A Danish study which conducted a three-year school-based PA intervention, surprisingly showed that doubling the amount of physical education from the first year of primary school had no significant effect on overall PA levels in the intervention group [30]. The study also emphasized that a possible explanation could be that children in the intervention group compensated for the increased school-time PA during the remainder of the day [30]. Another study, also conducted among primary school children, showed that PA participation during physical education classes and after-school was associated with children’s overall MVPA, but not with their sedentary behaviour [31]. In accordance, some studies have questioned whether children compensate for higher levels of activity during school hours by being less active after school [32,33]. A suggested hypothesis is that when an individual increases their PA in one domain, there is a compensatory change in another domain to maintain an overall stable level of PA [34,35].

Existing guidelines on PA for children are commonly expressed in terms of frequency, time, and intensity [36]. Recommendations based on a measure of step counts from accelerometers and pedometers are desired. Measuring step counts is becoming more common in research and may be a reasonable approximation of daily physical activity [36,37]. While it is recommended that children obtain 60 min of MVPA per day, which is suggested to be associated with approximate 10,000–14,000 daily steps in preschool children aged four to six years, there is no certain number of daily steps that cuts across all ages [37]. A systematic review aiming to identify, among existing guidelines, the optimal step-count cutoff for children and adolescents aged five to nineteen years found that existing guidelines ranged between 9000 and 14,000 steps per day for PA cohort studies [38]. However, due to the risk of methodological bias, none of the guidelines was endorsed. In total, 12,000 steps per day for children and adolescents were suggested by the study with the lowest risk of methodological bias [38]. In our study, children took approximately 13,000 step counts per day regardless of setting. This lies within the recommendations and may create less space to increase the physical activity level.

The strength of the current study is that the children’s PA levels were assessed objectively with accelerometers for 24 h, thus limiting some of the biases associated with self-reported measures such as recall difficulties. Another strength is the use of the pre-post design where the children served as their own controls; this limits the risk of potential confusion. The optimal study would have been a randomized controlled trial; however, it would be extremely difficult to implement and recruit participants for a randomized study, as it essentially means that parents would have to give up their free choice of kindergarten type.

Certain limitations in this study must be considered. The SENS motion^®^ activity accelerometer has only been recognized as a technically reliable instrument to objectively capture daily step counts among hospitalized patients or patients with knee osteoarthritis [24,39] and has not been validated among children.

We had information on the type of transportation to and from the kindergarten; however, this was not divided into the two weeks (the outdoor setting or the conventional setting weeks). We cannot exclude the possibility that the observed compensation seen outside kindergarten time during the outdoor weeks could be due to changed transportation form to and from kindergarten. It may be that parents choose to drive their children to kindergarten during the outdoor week. However, because the majority of children in Copenhagen live close to their kindergarten, we do not expect this to be very different in this study. The parents reported that around 60% of the children walked or biked to kindergarten, which will probably be the same in the two weeks. Therefore, we do not believe the transportation form will differ much between the two weeks.

Furthermore, it would also have been relevant to have information about the size of the kindergarten and the outdoor area including geographical information, because differences in space to move around in the two environments may have had an impact on the step counts taken during time spent in the kindergarten. In a qualitative study performed in the included kindergartens, we found that there were more opportunities for children to move more versatilely in the outdoor environment and there were greater opportunities for gross motor games and risky games in the outdoor than in the conventional setting [40]. This may indicate that greater space has an impact on step counts.

We did not assess the PA or attitudes towards PA among the preschool teachers. However, preschool teachers’ individual attitudes and behaviours towards PA may play an important role in promoting PA among preschool children (19). A study from Norway that explored accelerometer-assessed associations between preschool teachers’ and children’s levels of MVPA during preschool hours, demonstrated an association between preschool teacher’s aggregated PA levels and four-to-six-year-olds’ individual PA levels (20). However, because we conducted a pre-post design where the same group of kindergarten teachers were both in the outdoor and the conventional setting, we do not suspect that kindergarten teachers’ attitudes towards PA would have influenced the results in this present study. Similarly, we did not assess the PA or attitudes towards PA among the parents. We did, however, ask if parents were active with their children and discovered that 80.7% of the parents were active with their child. Research indicates a strong correlation between parental and child PA behaviours [41] and it may be that parental attitude towards PA may influence the compensation seen among the children during the week in the outdoor kindergarten setting.

Methodological limitations, such as selection bias, may arise because of missing activity measures or being excluded from analysis due to incomplete data on physical activity. Moreover, a lack of statistical power may have reduced the chances of detecting the true effects. Thus, with a larger sample size we might have been able to show a significant association in total daily step counts.

It is more common in Denmark to have rotating kindergartens in larger cities compared to smaller cities; however, outdoor kindergartens which roughly can be compared to when the children are in the outdoor setting in rotating kindergartens can be found in all areas. We have, though, in our previous study shown that parents in the two largest cities in Denmark choosing outdoor kindergartens differ with respect to socio-demographics as compared to parents from the same area choosing conventional kindergartens [23]. Thus, caution should be taken when generalizing the results on a national level.

Data collection for the present study was paused due to the COVID-19 lockdown from 13 March until September 2020. Post-lockdown, the new situation may have changed the way the kindergarten teachers used the outdoor space, since it was recommended that children spend more time outdoor due to the lower infection risk. This could have influenced the children’s activity levels, leading to a smaller difference in steps taken between the two settings post-lockdown. However, we found no evidence of effect modification by enrolment date (before/after the Danish COVID-19 lockdown), though it should be mentioned that the number of children included pre-lockdown was low (n = 21).

## 5. Conclusions

Findings from this study suggest that children are more physically active during kindergarten hours in outdoor kindergartens compared to conventional kindergartens, but this may at best lead to a marginally higher total daily activity level, perhaps indicating some degree of subsequent compensation or more sedentary activity due to transportation to and from the outdoor setting. Findings from this study indicate that a supportive environment may be a potential trigger for healthy PA behaviour in everyday life and especially that targeting behaviour changes through structure community intervention, such as outdoor kindergartens, may be beneficial.

## Figures and Tables

**Table 1:** Characteristics of the included participants. Results presented as percentages unless otherwise stated.

	n *	%
Child age (years) (mean, sd)	55	5 (1)
Gender	63	
Boys	22	34.9%
Girls	41	65.1%
Ethnicity	63	
Western	55	87.3%
Non-Western	8	12.7%
Sport activity outside kindergarten		
Yes	30	48.4
No	32	51.6
Transportation form to/from kindergarten **		
Passive	25	39.7
Active	38	60.3
Parental employment status	63	
Non-employed	4	6.3
Employed	59	93.7%
Sports activity (parents)		
Yes	37	59.7
No	24	38.7
Not reported	1	1.6
Active together with children		
Yes	50	80.7
No	12	19.3

* Number of participants varies due to missing information. ** Active transport is defined as walking, biking or using a scooter to kindergarten. Passive transport is defined as being transported by car, bus, train, cargo bike or by sitting on the back of a bicycle.

**Table 2:** Difference in physical activity behaviour between the outdoor setting and the conventional setting (n = 74).

	Outdoor Setting Mean (SD)	Conventional Setting Mean (SD)	Mean Difference	95% CI	*p*-Value
Step counts per day	13,253 (3205)	12,726 (2896)	527	(−61; 1115)	0.08
Step counts during kindergarten time	6630 (2010)	5541 (1586)	1089	(669; 1508)	<0.0001
Step counts outside kindergarten time	6623 (1708)	7185 (1704)	−562	(−952; −171)	<0.01

**Table 3:** Comparison of children included and children not included in this study.

	N	Included %	N	Not Included %	*p*-Value
CHILDREN					
Gender					
Girls	22	65.1	35	42.6	0.01
Boys	41	34.9	26	57.4	
Ethnicity					
Western	55	87.3	58	95.1	0.23
Non-Western	8	12.7	3	4.92	
Sport activity					
Yes	30	48.4	32	52.5	0.65
No	32	51.6	29	47.5	
Transportation form					
Passive	38	60.3	10	65.6	0.55
Active	25	39.7	21	34.4	
PARENTS					
Education level					
Short	8	12.7	3	4.9	0.29
Medium	17	27.0	20	32.8	
Long	38	60.3	38	60.3	
Sports activity					
Yes	37	59.7	34	55.7	0.91
No	24	38.7	26	42.6	
Not reported	1	1.6	1	1.6	
Active together with children					
Yes	50	80.7	50	82.0	0.85
No	12	19.3	11	18.0	

## Data Availability

The data presented in this study are available on request from the corresponding author.
